# The impact of a preoperative nurse-led orientation program on postoperative delirium after cardiovascular surgery: a retrospective single-center observational study

**DOI:** 10.1186/s40560-023-00666-3

**Published:** 2023-05-17

**Authors:** Ryo Nakamura, Kyohei Miyamoto, Kaori Tsuji, Kana Ozaki, Hideki Kunimoto, Kentaro Honda, Yoshiharu Nishimura, Seiya Kato

**Affiliations:** 1 Department of Emergency and Critical Care Medicine, Wakayama Medical University, 811-1 Kimiidera, Wakayama City, Wakayama 641-8509 Japan; 2 Department of Thoracic and Cardiovascular Surgery, Wakayama Medical University, 811-1 Kimiidera, Wakayama City, Wakayama 641-8509 Japan; 3 Department of Nursing, Wakayama Medical University Hospital, 811-1 Kimiidera, Wakayama City, Wakayama 641-8510 Japan

**Keywords:** Postoperative delirium, Non-pharmacological approach, Cardiovascular surgery, Intensive care unit

## Abstract

**Background:**

Postoperative delirium in intensive care is common and associated with mortality, cognitive impairment, prolonged hospital stays and high costs. We evaluate whether a nurse-led orientation program could reduce the incidence of delirium in the intensive care unit after cardiovascular surgery.

**Methods:**

In this retrospective cohort study, we enrolled patients admitted to the intensive care unit for planned cardiovascular surgery between January 2020 and December 2021. A nurse-led orientation program based on a preoperative visit was routinely introduced from January 2021. We assessed the association between these visits and postoperative delirium in the intensive care unit. We also assessed predictors of postoperative delirium with baseline and intraoperative characteristics.

**Results:**

Among 253 patients with planned cardiovascular surgery, 128 (50.6%) received preoperative visits. Valve surgery comprised 44.7%, coronary surgery 31.6%, and aortic surgery 20.9%. Cardiopulmonary bypass use and transcatheter surgery were 60.5% and 12.3%, respectively. Incidence of delirium was lower in patients that received preoperative visits, and median hospital stay was shorter than in those without visits (18 patients [14.1%] vs 34 patients [27.2%], *P* < 0.01; 14 days vs 17 days, *P* < 0.01). After adjusting predefined confounders, preoperative visits were independently associated with decreased incidence of delirium (adjusted odds ratio [aOR] 0.45; 95% confidence interval [95% CI] 0.22–0.84). Other predictors of delirium were higher European System for Cardiac Operative Risk Evaluation II score and lower minimum intraoperative cerebral oxygen saturation.

**Conclusions:**

A preoperative nurse-led orientation program was associated with reduction of postoperative delirium and could be effective against postoperative delirium after cardiovascular surgery.

*Trial registration* UMIN Clinical Trial Registry no. UMIN000048142. Registered 22, July, 2022, retrospectively registered, https://center6.umin.ac.jp/cgi-open-bin/ctr/ctr_view.cgi?recptno=R000054862.

**Supplementary Information:**

The online version contains supplementary material available at 10.1186/s40560-023-00666-3.

## Background

Delirium is an acute and transient brain dysfunction that is common during intensive care unit (ICU) stays, with reported frequency of about 30% after cardiovascular surgery [1, 2]. Postoperative delirium is independently associated with mortality, long term cognitive impairment, prolonged hospital stay and higher medical cost [1–3], so prevention is important.

Regarding prevention of postoperative delirium, the pharmacological approach to delirium in ICU has been extensively explored in the literature (e.g. dexmedetomidine), but the efficacy has been controversial [4–6]. By contrast, two recent randomized controlled trials showed that non-pharmacological approaches may play important roles in preventing postoperative delirium. A bundle approach including staff education and postoperative intervention was reported to significantly reduce postoperative delirium in patients undergoing noncardiac surgery, but there was no such significant reduction in cardiac surgery [7]. The pathophysiology and potential interventions for delirium in cardiac surgery are suggested to potentially differ from those in general surgery. Preoperative cognitive prehabilitation reportedly reduced the incidence of postoperative delirium, but without statistically significant difference [8]. Such results suggest the promising role of nonpharmacological intervention, but more information is needed about nonpharmacological preventive strategy for patients receiving cardiac surgery.

Patients undergoing cardiac surgery often perceive anxiety preoperatively, which is associated with postoperative delirium [9]. Known as the stress coping theory, understanding the meaning and importance of a particular stressor could facilitate stress coping and alleviate any anxiety [10]. We hypothesized that postoperative delirium may be reduced or may even be preventable by implementation of a preoperative orientation program to reduce preoperative anxiety. In pediatric surgery, preoperative orientation including the use of a video and pamphlet reduced preoperative anxiety and the incidence of postoperative delirium [11]. With the aim of preventing or reducing the incidence of postoperative ICU delirium in adult patients, we introduced a similar preoperative orientation program to facilitate patient understanding of the situations and interventions that they would likely experience in ICU. This study evaluates whether a nurse-led preoperative orientation program could prevent or reduce ICU delirium in adult patients after cardiovascular surgery.

## Methods

### Study design and participants

**Fig. 1:**
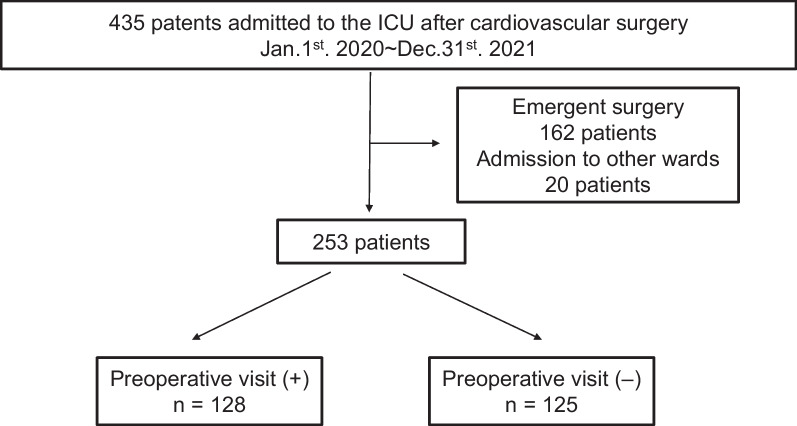
Patient flowchart

Enrolled in this study were 253 of the 435 consecutive adult patients (≥ 20 years old) who underwent cardiovascular surgery at the Wakayama Medical University Hospital between January 1st 2020 and December 31st 2021. Owing to the need for emergent surgery, 162 patients were excluded, and 20 patients were excluded because they were admitted to a department outside of ICU, such as to a cardiac care unit or general ward (Fig. 1).

### Ethical statement

This single-center retrospective cohort study was approved by Wakayama Medical University Institutional Review Board (approval number 3532) and was registered in the UMIN Clinical Trial Registry on 23rd June, 2022 (registration no. UMIN000048142). The need for informed consent was waived because of the retrospective and observational nature of this study, but the study was conducted in accordance with the principles of the Declaration of Helsinki.

### Intervention

**Fig. 2:**
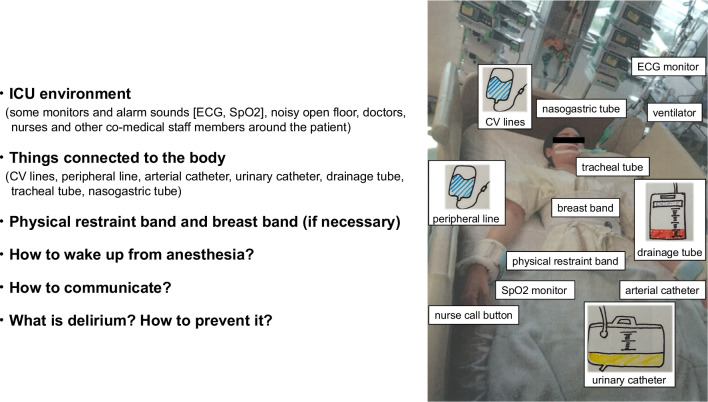
Example of orientation with figure used in the nurse-led orientation program. ECG: electrocardiogram; SpO_2_: peripheral saturation of oxygen; CV: central venous

**Fig. 3:**
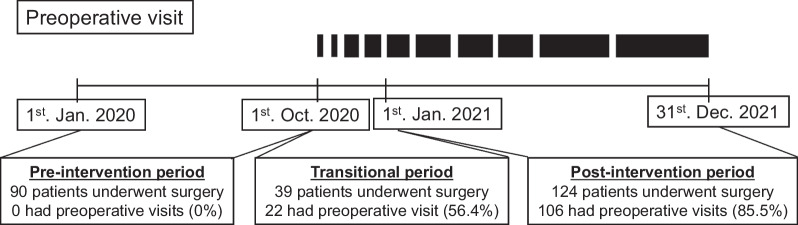
Intervention period. A nurse-led preoperative educational program (preoperative visit) was gradually introduced from October 2020, and used routinely from January 2021

We introduced a nurse-led preoperative orientation program as a preventative measure against delirium during postoperative ICU stays (Fig. 2). In this program, nurses specializing in ICU care visit the patients on the day before surgery. Initially, they introduce themselves and their work to the patients. Patients then receive orientation about the ICU environment which includes, for example, how to wake up from anesthesia in the ICU bed, the kinds of noises such as alarm sounds that they will hear in ICU, and about the medical staff members such as nurses and physicians that will be working around them during their stay there. The nurses then explain the procedures that patients will undergo (e.g. mechanical ventilation, central venous catheter, atrial catheter, chest drainage tube, etc.). The patient is informed that she/he cannot initially speak because of the use of tracheal tube, but they may communicate with nurses and physicians by gesture or writing. Patients are informed that they may be physically restrained. Finally, patients receive information about postoperative delirium (e.g. “what is delirium”, “what symptoms of delirium might patients have?”, or “what is the treatment for delirium?”) During this orientation visit, which takes approximately 30 min, nurses use a pamphlet including pictures and explanatory text. Wherever possible, nurses who will be responsible for providing care of the patient during their ICU stay participate in the preoperative orientation. This intervention, named ‘preoperative visit’, was transitionally introduced in October 2020 and then routinely performed from January 2021 (Fig. 3). The same preoperative orientations were used in the transitional period and post-intervention period.

In some cases, the preoperative visit was not conducted during the transitional or the post-intervention period because the patients were not available at the time of orientation visit for other preoperative examinations, etc. The patients’ selection of the intervention was not based on their preoperative factors.

### Outcomes

The primary outcome of this study was the occurrence of delirium, which was defined as at least one positive result on the Confusion Assessment Method for the ICU (CAM-ICU) during their ICU stay [12]. CAM-ICU was assessed daily by trained nurses during the daytime when patients were not under deep sedation, and there was also assessment of whether there was change in patients' mental status. Secondary outcomes included the length of ICU and hospital stay, hospital discharge to home, and medical costs during hospitalization.

### Procedures during surgery and ICU

In the operating room, general anesthesia was performed with propofol, remifentanil, sevoflurane, or rocuronium at the discretion of the anesthesiologist at the time. Additional epidural anesthesia by levobupivacaine or fentanyl was used for patients who received thoracoabdominal incision. INVOS system (Covidien, Minneapolis, MN) was routinely used to measure the regional cerebral oxygen saturation (rScO_2_) at the left and right forehead during the operation to monitor the appropriate cerebral perfusion. The value used was the mean rScO_2_ of the left and right forehead.

In our ICU, we performed the standard preventive measure for delirium known as the ABCDEF bundle [13]. Analgosedation was performed using fentanyl, propofol, and/or dexmedetomidine for patients receiving mechanical ventilation. Dexmedetomidine was used at the discretion of the physician responsible for sedation during mechanical ventilation and prevention of postoperative delirium. All patients were routinely evaluated for pain using the numeric rating scale or behavioral pain scale, as appropriate. Fentanyl and intravenous acetaminophen were mainly used to alleviate pain towards the goal of behavioral pain scale < 5 or numeric rating scale < 4. All patients receiving mechanical ventilation routinely underwent daily spontaneous awakening trials and spontaneous breathing trials as a means of weaning them from mechanical ventilation when it was considered to be appropriate. Sedation depth was evaluated using the Richmond Agitation Sedation Scale every 1–3 h. The goal of sedation in patients during the spontaneous awakening trial was set to Richmond Agitation Sedation Scale of 0 or − 1 (calm) during the day and − 2 or − 3 (lightly sedated) during the night. We avoided using benzodiazepines, a known cause of delirium, and we evaluated the occurrence of delirium with CAM-ICU, mentioned in the outcome subsection below. We routinely performed early mobilization and rehabilitation after the surgery. Before the COVID-19 pandemic, family visitation was allowed in accordance with the requests of patients and their families.

### Statistical analysis

Continuous variables are presented as median and interquartile range (IQR) or average and standard deviation. Categorical variables are presented as numbers and percentages. To compare two groups, Student’s t test or Wilcoxon rank sum test were used for continuous variables, and Pearson’s chi-square test or Fisher’s exact test were used for categorical variables, as appropriate. Univariate and multivariate logistic regression models were used to assess the association between the preoperative orientation visits and delirium. The multivariate logistic model used predefined adjusters that were selected because previous literature and clinical judgement suggested they would be risk factors of postoperative delirium [1, 14]. These adjusters included age (≥ 75 years, median), history of cerebrovascular and mental illness (at least one of cerebrovascular disease, mental illness, history of delirium during previous hospitalization, and dementia), use of cardiopulmonary bypass (CPB), operation time, intubation time after surgery (≥ 14 h, median), and perioperative platelet cell transfusion received. As sensitivity analysis for primary results and as exploratory analysis to examine risk factors on postoperative delirium, we separately constructed univariate and multivariate logistic regression models using variables from baseline characteristics (age, gender, body mass index [BMI], left ventricular ejection fraction, chronic kidney disease (estimated glomerular filtration rate < 60 ml/min/1.73 m^2^), respiratory disorder (forced expiratory volume 1.0% < 70% and/or % vital capacity < 80% defined by spirometry), diabetes mellitus, history of smoking and any cerebrovascular or mental illness), the European System for Cardiac Operative Risk Evaluation II (EuroSCORE II), and characteristics during operation (type of surgery, operation time, CPB use, rScO_2_, any transfusion received, and highest plasma lactate level). The multivariate model used variables that were *P* value < 0.05 in the univariate model. Additionally, we conducted the abovementioned analyses but with exclusion of the transitional period for the purpose of more clearly understanding sensitivity, because this period had a mixture of patients that received preoperative visits and patients that did not.

JMP Pro software (version 16; SAS Institute, Cary, NC) was used in all analyses. A two-sided *P* value < 0.05 was considered to be statistically significant.

## Results

During the transitional period, 22 of 39 postoperative patients (56.4%) received preoperative visits, and 106 of 124 postoperative patients (85.5%) received visits during the post-intervention period (Fig. 3). Among all enrolled patients, 128 (50.6%) received preoperative visits (preoperative visit group), and 125 (49.4%) did not (non-preoperative visit group). We evaluated the CAM-ICU for the detection of delirium in all patients and found no missing values.

Baseline, intraoperative, and postoperative characteristics are shown in Table 1. In the group of patients that received preoperative visits, more patients had restrictive respiratory pattern detected by spirometry and experienced longer CPB time than in the group that did not receive preoperative visits. There was no statistically significant difference in other variables between the two groups.

**Table 1:** Patient characteristics

Characteristics	Preoperative visit (+) (n = 128)	Preoperative visit (–) (n = 125)	*P* value
*Characteristics at baseline*			
Age, y, median (IQR)	74 (69–80)	75 (69–79)	0.82
Male, n (%)	81 (63.3)	82 (65.6)	0.70
Body mass index (kg/m^2^) Mean ± SD	23.0 ± 3.8	23.0 ± 3.8	0.48
Left ventricular ejection Fraction < 50%, n (%)	35 (27.3)	33 (26.4)	0.86
Chronic kidney disease (eGFR < 60), n (%)	80 (62.5)	80 (64.0)	0.80
Chronic hemodialysis, n (%)	10 (7.8)	16 (12.8)	0.19
FEV1.0% < 70%, n (%)^b^	24 (18.8)	23 (18.4)	0.94
**%VC < 80%, n (%)**^b^	47 (37.7)	74 (59.2)	< 0.01
Diabetes mellitus, n (%)	45 (35.2)	44 (35.2)	0.99
History of smoking, n (%)	62 (48.4)	48 (38.4)	0.11
Cerebrovascular disease, n (%)	17 (13.3)	16 (12.8)	0.91
Psychiatric disorder, n (%)	2 (1.6)	1 (0.8)	1.00
Dementia, n (%)	1 (0.8)	2 (1.6)	0.62
History of delirium, n (%)	2 (1.6)	0 (0)	0.50
Any of cerebrovascular and mental illness, n (%)	21 (16.4)	18 (14.4)	0.66
Chronic benzodiazepines use	15 (11.7)	11 (8.8)	0.44
Chronic anti-psychiatric medications use	3 (2.3)	1 (0.8)	0.62
Chronic opioid use	1 (0.8)	0 (0)	1.0
Previous cardiac surgery, n (%)	13 (10.2)	10 (8.0)	0.55
EuroSCORE II	3.2 (2.08–5.44)	2.7 (1.71–4.81)	0.09
*Characteristics during surgery*			
Main surgery			0.97
Coronary, n (%)	40 (31.3)	40 (32.0)	
Valve, n (%)	57 (44.5)	56 (44.8)	
Aorta, n (%)	28 (21.9)	25 (20.0)	
Others, n (%)	3 (2.3)	4 (3.2)	
Concomitant surgery, n (%)	36 (28.1)	42 (33.6)	0.36
Transcatheter surgery, n (%)	18 (14.1)	13 (10.4)	0.37
Operation time, min, mean ± SD	285 ± 103	287 ± 97	0.89
Cardiopulmonary bypass, n (%)	78 (60.9)	75 (60.0)	0.88
**Cardiopulmonary bypass time, min, median (IQR)**	179 (149–215)	159 (136–187)	0.026
Selective cerebral perfusion, n (%)	13 (16.7)	13 (17.3)	0.95
rScO_2_ minimum^a^, %, median (IQR)	54 (47.0–61.0)	54 (50.0–59.0)	0.96
*Characteristics during ICU care*			
Mechanical ventilator use, n (%)	120 (93.8)	119 (95.2)	0.61
Intubation time, hour, median (IQR)	14 (8.5–18.0)	15 (9.0–21.0)	0.21
Length of ICU stay, hour, median (IQR)	22 (19.0–43.0)	22 (19.0–67.0)	0.29
Propofol use, n (%)	119 (93.0)	118 (94.4)	0.64
**Dexmedetomidine use, n (%)**	31 (24.2)	47 (36.8)	0.030
Benzodiazepine use, n (%)	0 (0)	0 (0)	1.00
Antipsychotic medication use, n (%)	0 (0)	0 (0)	1.00
Dopamine use, n (%)	96 (75.0)	103 (82.4)	0.15
Dobutamine use, n (%)	36 (28.1)	30 (24.0)	0.46
Noradrenaline use, n (%)	32 (25.0)	40 (32.0)	0.22
RCC transfusion, n (%) (including during surgery)	94 (73.4)	90 (72.0)	0.80
FFP transfusion, n (%) (including during surgery)	70 (54.7)	71 (56.8)	0.74
PC transfusion, n (%) (including during surgery)	55 (43.0)	53 (42.4)	0.93
Highest plasma lactate during ICU stay, mmol/L, median (IQR)	2.8 (1.4–4.4)	2.8 (1.6–4.0)	0.97
Postoperative renal replacement therapy, n (%)	13 (10.2)	18 (14.4)	0.30
Perioperative atrial fibrillation, n (%) (including chronic atrial fibrillation)	22 (17.2)	21 (16.8)	0.93
APACHEII score, median (IQR)	16 (13–18)	16 (13–19)	0.22

APACHE II: Acute Physiology and Chronic Health Evaluation II; eGFR: estimated glomerular filtration rate; EuroSCORE II: The European System for Cardiac Operative Risk Evaluation II; FEV1.0%: forced expiratory volume % in one second; FFP: fresh frozen plasma; ICU: intensive care unit; IQR: interquartile range; PC: platelet concentrate; RCC: red cell concentrate; rScO_2_: regional cerebral oxygen saturation; SD: standard deviation; %VC: % vital capacity

^a^rScO_2_ minimum data were missing for two patients in preoperative visit (–) and one patient in preoperative visit (+)

^b^Respiratory spirometry test (FEV1.0% and %VC) were missing for three patients in preoperative visit (–) and two patients in preoperative visit (+)

Regarding outcomes (Table 2), the preoperative visit group had lower occurrence rate of postoperative delirium than the non-preoperative visit group (18 patients [14.1%] vs 34 patients [27.2%], *P* < 0.01). Absolute difference of delirium occurrence observed in our study was 13.1%, and the number requiring treatment to prevent additional postoperative delirium was approximately 7. The time from surgery to the occurrence of delirium did not differ between the two groups (1 day [IQR: 1–1] vs 1 day [1–2.5], *P* = 0.23). Length of hospital stay was significantly shorter in the preoperative visit group than that in non-preoperative visit group (14 days vs 17 days, *P* < 0.01). In the multivariate logistic regression model with adjustment for the influence of predefined confounders, there was also significant association between preoperative visit and postoperative delirium (adjusted odds ratio [aOR] 0.45; 95% confidence interval [CI] 0.22–0.84, *P* = 0.013). Similar results were obtained in the analysis after exclusion of patients in the transitional period (Additional file 1: Table S1).

**Table 2:** Outcome

Characteristics	Preoperative visit (+) (n = 128)	Preoperative visit (–) (n = 125)	*P* value
*Primary outcome*			
**Delirium, n (%)**	18 (14.1%)	34 (27.2%)	< 0.01
*Secondary outcome*			
Length of ICU stay, day, Median (IQR)	1 (1–2)	1 (1–3)	0.067
**Length of hospital stay, day, Median (IQR)**	14.0 (11.0–20.8)	17.0 (13.0–23.0)	< 0.01
Hospital death, n (%)	3 (2.3)	1 (0.8)	0.62
Discharge to home, n (%)	104 (81.3)	92 (74.2)	0.083
Medical cost, million yen, Median (IQR)	5.04 (3.92–6.59)	5.18 (3.66–6.97)	0.96

ICU: intensive care unit; IQR: interquartile range

The results of sensitivity analysis that separately explore the risk factors on postoperative delirium are shown in Table 3. In the multivariate logistic regression model, independent predictors were preoperative visit (aOR 0.29; 95% CI 0.13–0.64, *P* < 0.01), previous cardiac surgery (aOR 3.08; 95% CI 1.06–8.97, *P* = 0.039), EuroSCORE II (aOR 1.21; 95% CI 1.06–1.38, *P* < 0.01), and minimum rScO_2_ during surgery (aOR 0.96; 95% CI 0.92–0.99, *P* = 0.032). Similar results were obtained in the analysis after exclusion of patients in the transitional period (Additional file 2: Table S2).

**Table 3:** Predictors for postoperative delirium

	Univariable OR (95% CI)	*P* value	Multivariable OR (95% CI)	*P* value
**Preoperative visit**	0.44 (0.23–0.83)	0.011	0.29 (0.13–0.64)	< 0.01
*Characteristics at baseline*				
Age ≥ 75 years old	1.56 (0.84–2.87)	0.16		
Male	0.57 (0.31–1.06)	0.076		
Body mass index per 1 point increase	0.88 (0.80–0.97)	< 0.01	0.92 (0.82–1.02)	0.11
Left ventricular ejection fraction < 50%	1.99 (1.04–3.80)	0.037	1.56 (0.72–3.38)	0.26
Chronic kidney disease (eGFR < 60)	2.25 (1.11–4.55)	0.024	0.93 (0.38–2.28)	0.88
FEV1.0 < 70%^a^	2.44 (1.21–4.92)	0.013	1.47 (0.64–3.37)	0.36
%VC < 80%^a^	1.65 (0.89–3.05)	0.11		
Diabetes mellitus	0.97 (0.51–1.84)	0.92		
History of smoking	1.04 (0.56–1.92)	0.90		
Any cerebrovascular or mental illness	1.66 (0.76–3.60)	0.20		
**Previous cardiac surgery**	5.18 (2.14–12.6)	< 0.01	3.08 (1.06–8.97)	0.039
**EuroSCORE II per 1 point increase**	1.27 (1.15–1.41)	< 0.01	1.21 (1.06–1.38)	< 0.01
*Characteristics during surgery*				
Median full sternotomy	2.07 (0.87–4.89)	0.096		
Transcatheter surgery	0.92 (0.36–2.37)	0.86		
Operation time per 1 h increase	1.28 (1.06–1.54)	< 0.01	1.17 (0.95–1.46)	0.14
Cardiopulmonary bypass use	1.81 (0.93–3.50)	0.080		
**rScO**_2_ **minimum per 1% increase**^b^	0.95 (0.92–0.98)	< 0.01	0.96 (0.92–0.99)	**0.032**
Transfusion received	2.45 (1.09–5.52)	0.030	0.99 (0.36–2.77)	0.99
Highest plasma lactate level ≥ 2.0 mmol/l	1.71 (0.92–3.18)	0.088		

eGFR: estimated glomerular filtration rate; EuroSCORE II: The European System for Cardiac Operative Risk Evaluation II; FEV1.0%: forced expiratory volume % in one second; ICU: intensive care unit; rScO_2_: regional cerebral oxygen saturation; %VC: % vital capacity

^a^Three patients in preoperative visit (–) and two patients in preoperative visit (+) were not evaluated in preoperative pulmonary function test because they had no problems in their physical activities. We considered these patients' results of spirometry to be normal in logistic regression model

^b^Regional cerebral oxygen saturation (rScO2) at the forehead was not measured in two patients in preoperative visit (–) and one patient in preoperative visit (+), they were treated as missing values in multivariate logistic regression model

## Discussion

Our nurse-led preoperative orientation program (preoperative visit) as a non-pharmacological approach for reducing/preventing delirium was associated with reduced incidence of postoperative delirium in cardiovascular surgery. Furthermore, the preoperative visit was associated with shorter length of hospital stay, but not associated with length of ICU stay, in-hospital mortality, discharge to home, or medical cost during hospitalization. Additionally, three risk factors of postoperative delirium were identified as previous cardiac surgery, higher EuroSCORE II and lower rScO_2_ during surgery.

Preoperative mental unfitness is considered to be part of postoperative delirium pathophysiology [9]. A prospective cohort study of 306 patients that underwent coronary artery bypass graft surgery reported that half of patients perceived anxiety preoperatively, and these patients had higher incidence of postoperative delirium than those without preoperative anxiety (31.3% vs. 7.8%) [9]. A randomized controlled trial that enrolled 408 pediatric patients that underwent general surgery reported that preoperative intervention using videos and pamphlets to help patients understand their own postoperative situation significantly reduced the intensity of preoperative anxiety, and further, reduced the incidence of postoperative delirium compared with a control group (10.4% vs. 24.2%) [11]. The preoperative orientation program in our study might also reduce the incidence of postoperative delirium by reducing preoperative anxiety, but this remains unclear because there was no evaluation of preoperative anxiety. This should be elucidated in future prospective studies.

There are pharmacological and non-pharmacological approaches to preventing delirium, and some randomized controlled trials have shown the effectiveness of non-pharmacological interventions in reducing postoperative delirium in non-cardiovascular surgery. A stepped-wedge cluster randomized clinical trial showed that a delirium prevention program in older patients was effective in reducing the incidence of postoperative delirium [7]. This delirium prevention program consisted of structured education on delirium for clinical caregivers and the seven best-practice delirium prevention modules (cognitive, motor, and sensory stimulation; meal companionship; diagnostic test and operating room accompaniment; stress relaxation; sleep promotion), but it did not include preoperative intervention. The program was shown to be effective in orthopedic and general surgery, but not in cardiac surgery. Cardiovascular surgery is one of the most invasive types of surgery, especially when there is CPB. Unlike non-cardiac surgery, most patients receiving cardiac surgery are admitted to ICU postoperatively and experience extensive change in environment. In cardiovascular surgery, these differences are expected to affect patients' cognitive function such as disorientation, and this problem requires other preventive measures, for instance a preoperative orientation program, which was evaluated in our study.

As another non-pharmacological approach in cardiovascular surgery, a team-based approach including educational sessions for the medical team and bundle approach for insomnia and agitation (avoiding the use of benzodiazepine) were reportedly associated with a lower incidence of postoperative delirium and reduced the length of hospital stay [15]. Such postoperative intervention (team-based approach) could be combined with the preoperative intervention in our study (preoperative visit) to potentially bring further reduction of postoperative delirium in cardiovascular surgery, although this requires confirmation in future studies.

Regarding pharmacological perspectives, certain drugs may affect the incidence of postoperative delirium. Benzodiazepines are strongly associated with delirium [16], and dexmedetomidine may have a protective effect against delirium [5, 6]. In our study, no patients received benzodiazepine, while more patients in the non-preoperative visit group received dexmedetomidine than in the preoperative visit group during ICU stay. If more patients had received dexmedetomidine for delirium prevention in the non-preoperative visit group, it might decrease the incidence of delirium in that group. In reality, however, the observed incidence of delirium was higher in non-preoperative visit group. This might reinforce our hypothesis that preoperative visits could prevent delirium. However, it is possible that more patients received dexmedetomidine for delirium treatment as a result of increased delirium occurrence.

This exploratory study identified risk factors; aside from preoperative visit, previous cardiac surgery, higher EuroSCORE II and lower rScO_2_ during surgery were associated with postoperative delirium. Their relationship with delirium has been previously reported [17, 18]. In general, surgery in a patient who has undergone previous cardiac surgery is associated with longer operation time and more invasive surgery than in patients without previous cardiac surgery. This results in higher risk of postoperative delirium. Preoperative risk assessment using EuroSCORE II in patients who have undergone previous cardiac surgery may be a key to predicting postoperative delirium. The relationship between rScO_2_ and postoperative delirium has been reported: lower intraoperative rScO_2_ was reported to be associated with postoperative delirium in endovascular surgery [19], and off-pump coronary artery bypass grafting surgery [20]. These findings were concordant with the results of our study. Maintaining rScO_2_ during surgery could be a preventive factor against postoperative delirium. Future studies will seek to confirm this.

### Limitations

This study has several limitations. The most critical limitation is the retrospective nature of this study in the way that it evaluates the use of transitionally-introduced intervention in the form of a preoperative visit. The association between delirium and intervention may have been confounded by treatment or institutional change other than the preoperative visit program. For example, due to the coronavirus disease 2019 (COVID-19) pandemic, restrictions on family visits were introduced in our institute in September 2020, which was almost simultaneous with the introduction of preoperative visits. Family visitation restrictions have been reported to be associated with increase of delirium [21, 22]. Preoperative visits, which were almost simultaneously introduced with family visitation restriction, were nevertheless still associated with the reduced incidence of postoperative delirium in our study. This reinforces rather than weakens the hypothesis that preoperative visits have a delirium-preventive effect. No other major treatment or institutional changes occurred during the study period, although minor differences in sedation management and attitude change might occur because of its nature of retrospective study. Furthermore, to adjust the influence of confounders, we carefully selected adjusters used in a multivariate model from previous studies or clinical experiences in advance. Additionally, sensitivity analysis to confirm the primary analysis showed similar results, which assured the robustness of the study results. Second, this is a retrospective study, and outcomes were not evaluated prospectively. To evaluate the primary outcome, we used CAM-ICU to diagnose postoperative delirium. The retrospective nature of our study did not allow us to evaluate the influence of using other diagnostic tools (e.g. Intensive Care Delirium Screening Checklist) other than CAM-ICU to diagnose delirium. However, CAM-ICU was an objective, broadly-used, and well-validated tool to define delirium in the literature, and was used in daily clinical practice by trained nurses in our ICU. The influence of retrospective design on outcome evaluation therefore seems to be minimal in our study. A third limitation is that this is a single center study, so generalizability cannot be guaranteed, and a multicenter study is required to confirm these results. In summary, this can be considered a hypothesis-generating study because of the limitations. Future prospective studies will seek to validate our results.

## Conclusion

A preoperative nurse-led orientation program was associated with decreased incidence of postoperative delirium in our cohort of patients undergoing cardiovascular surgery. A similar intervention could be applied as a single intervention or as an element of a bundle approach with other pharmacological or non-pharmacological approaches. Studies will seek to confirm whether such intervention could be an effective preventative measure against postoperative delirium in similar patients.

## Supplementary Information

**Additional file 1:** **Table S1.** Outcomes except for transitional period.

**Additional file 2:** **Table S2.** Predictors for postoperative delirium except for transitional period.

## Data Availability

The data underlying this article will be shared on reasonable request to the corresponding author.

## Abbreviations

aOR: Adjusted odds ratio
BMI: Body mass index
CAM-ICU: Confusion assessment method for the intensive care unit
CI: Confidence interval
COVID-19: Coronavirus disease 2019
CPB: Cardiopulmonary bypass
EuroSCORE II: The european system for cardiac operativerisk evaluation II
ICU: Intensive care unit
IQR: Interquartile range
rScO2: Regional cerebral oxygen saturation
UMIN: University hospital medical information network
