# Supplementary material for: The impact of a preoperative nurse-led orientation program on postoperative delirium after cardiovascular surgery: a retrospective single-center observational study
Source: J Intensive Care. 2023 May 17;11:20. doi: 10.1186/s40560-023-00666-3 (PMC10191397; doi:10.1186/s40560-023-00666-3)
Supplement: Supplementary file 1 — Additional file 1: Table S1. Outcomes except for transitional period. [file 40560_2023_666_MOESM1_ESM.docx]

Table S1. Outcome (except for transitional period)

| Characteristics | Preoperative visit (+)  (n = 106) | Preoperative visit (–)  (n = 108) | *P* value |
| --- | --- | --- | --- |
| **Primary Outcome** | | | |
| **delirium, n (%)** | **18 (14.2%)** | **29 (26.9%)** | **0.021** |
| **Secondary Outcome** | | | |
| length of ICU stay, hour, median (IQR) | 21.5 (19.0–43.3) | 22.0 (19.3–67.0) | 0.27 |
| **length of Hospital stay, day, median (IQR)** | **14.0 (10.0–22.3)** | **17.0 (13.0–23.8)** | **< 0.01** |
| hospital death, n (%) | 3 (2.8) | 1 (0.9) | 0.37 |
| discharge to home, n (%) | 84 (81.6) | 79 (73.8) | 0.18 |
| medical cost, million yen, median (IQR) | 5.20 (3.98–6.76) | 5.16 (3.66–6.93) | 0.60 |

ICU: intensive care unit, IQR: interquartile range
