# Supplementary material for: The impact of a preoperative nurse-led orientation program on postoperative delirium after cardiovascular surgery: a retrospective single-center observational study
Source: J Intensive Care. 2023 May 17;11:20. doi: 10.1186/s40560-023-00666-3 (PMC10191397; doi:10.1186/s40560-023-00666-3)
Supplement: Supplementary file 2 — Additional file 2: Table S2. Predictors for postoperative delirium except for transitional period. [file 40560_2023_666_MOESM2_ESM.docx]

Table S2. Predictors for postoperative delirium (except for transitional period)

|  | Univariable OR (95% CI) | *P* value | Multivariable OR (95% CI) | *P* value |
| --- | --- | --- | --- | --- |
| **preoperative visit** | **0.45 (0.22–0.90)** | **0.021** | **0.25 (0.10–0.63)** | **< 0.01** |
| characteristics at baseline | | | | |
| age ≥75 years old | 1.52 (0.78–2.96) | 0.22 |  |  |
| male | 0.54 (0.28–1.07) | 0.073 |  |  |
| **body mass index per 1 point increase** | **0.87 (0.79–0.97)** | **< 0.01** | 0.93 (0.82–1.05) | 0.21 |
| **left ventricular ejection fraction < 50%** | **1.99 (1.04–3.80)** | **0.035** | 1.61 (0.67–3.91) | 0.29 |
| chronic kidney disease (eGFR < 60) | 2.00 (0.95–4.23) | 0.080 |  |  |
| **FEV1.0 < 70%*** | **3.16 (1.51–6.65)** | **< 0.01** | 1.81 (0.74–4.41) | 0.19 |
| **%VC < 80%*** | **1.97 (1.01–3.87)** | **0.045** | 1.03 (0.45–2.33) | 0.95 |
| diabetes mellitus | 0.86 (0.46–1.86) | 0.82 |  |  |
| history of smoking | 1.15 (0.59–2.24) | 0.68 |  |  |
| any cerebrovascular or mental illness | 1.29 (0.54–3.09) | 0.57 |  |  |
| **previous cardiac surgery** | **5.42 (2.20–13.36)** | **< 0.01** | **3.15 (1.06–9.34)** | **0.038** |
| **EuroSCORE** II per 1 point increase | **1.29 (1.16-1.43)** | **< 0.01** | **1.24 (1.08–1.42)** | **< 0.01** |
| characteristics during surgery | | | | |
| median full sternotomy | 2.17 (0.80–5.90) | 0.13 |  |  |
| transcatheter surgery | 0.79 (0.26–2.47) | 0.69 |  |  |
| **operation time (hour)** | **1.30 (1.06–1.59)** | **< 0.01** | 1.06 (0.81–1.38) | 0.69 |
| **cardiopulmonary bypass use** | **2.20 (1.04–4.65)** | **0.038** | 1.26 (0.43–3.71) | 0.68 |
| **rScO_2_ minimum per 1% increase** | **0.95 (0.92–0.98)** | **< 0.01** | 0.96 (0.92–1.00) | 0.073 |
| **transfusion received** | **2.79 (1.11–7.01)** | **0.024** | 0.87 (0.26–2.95) | 0.83 |
| **highest plasma lactate level ≥2.0 mmol/l** | **1.98 (1.01–3.88)** | **0.044** | 1.55 (0.61–3.98) | 0.36 |

* Three patients in preoperative visit (–) and two patients in preoperative visit (+) were not evaluated in preoperative pulmonary function test because they had no problems in their physical activities. We considered these patients' results of spirometry to be normal in logistic regression model.

§ Regional cerebral oxygen saturation (rScO2) at the forehead was not measured in one patients in preoperative visit (–), and he was treated as missing value in multivariate logistic regression model.

eGFR: estimated glomerular filtration rate, EuroSCORE II: The European System for Cardiac Operative Risk Evaluation II, FEV1.0%: forced expiratory volume % in one second, ICU: intensive care unit, rScO_2_: regional cerebral oxygen saturation, %VC: % vital capacity
